# Porocarcinoma: Clinical and Histological Features, Immunohistochemistry and Outcomes: A Systematic Review

**DOI:** 10.3390/ijms25115760

**Published:** 2024-05-25

**Authors:** Thomas Bienstman, Canan Güvenç, Marjan Garmyn

**Affiliations:** 1 Department of Dermatology, University Hospitals Leuven, 3000 Leuven, Belgium; 2 Department of Oncology, Laboratory of Dermatology, Catholic University of Leuven, 3000 Leuven, Belgium

**Keywords:** porocarcinoma, adnexal tumor, sweat gland tumor, immunohistochemistry, Mohs micrographic surgery

## Abstract

Porocarcinoma (PC) is a rare adnexal tumor, mainly found in the elderly. The tumor arises from the acrosyringium of eccrine sweat glands. The risk of lymph node and distant metastasis is high. Differential diagnosis with squamous cell carcinoma is difficult, although NUT expression and YAP1 fusion products can be very useful for diagnosis. Currently, wide local excision is the main surgical treatment, although Mohs micrographic surgery is promising. To date, there is no consensus regarding the role of sentinel lymph node biopsy and consequential lymph node dissection. No guidelines exist for radiotherapy, which is mostly performed based on tumor characteristics and excision margins. Only a few studies report systemic treatment for advanced PC, although therapy with pembrolizumab and EGFR inhibitors show promise. In this review, we discuss epidemiology, clinical features, histopathological features, immunohistochemistry and fusion products, surgical management and survival outcomes according to stage, surgical management, radiotherapy and systemic therapy.

## 1. Introduction

Porocarcinoma (PC) is a malignant appendageal tumor which is thought to arise from the intra-epidermal part of the eccrine sweat glands (acrosyringium). Malignant eccrine poroma and malignant hidroacanthoma simplex are synonyms frequently used in the literature, although the use of the preposition malignant with a benign counterpart is not preferable. Porocarcinoma can arise in a pre-existing poroma or occur de novo [1]. Malignant transformation can occur after up to 8.5 years, and studies suggest sudden growth, bleeding or ulceration in a pre-existing poroma are suggestive of malignant transformation [1,2].

Porocarcinoma is a rare tumor, only representing 0.005–0.01% of cutaneous neoplasms. Overall, incidence rates are in the range of 0.02–0.2 per 100,000 person years [3]. In general, PC is more common in the elderly population, most commonly in patients 70–80 years old. Other risk factors are chronic immunosuppression and possibly chronic ultra-violet light or pesticide exposure [1]. Morphologically, PC usually presents as a solitary, indurated papule or nodule. Porocarcinoma can occur on the lower extremities and the head and neck region, but which site is most frequent remains elusive [1]. Diagnosis can be challenging since PC can mimic squamous cell carcinoma (SCC) or other cutaneous adnexal malignancies (CAM) clinically and histologically [3]. Immunohistochemical markers carcinoembryogenic antigen (CEA) and epithelial membrane antigen (EMA) are frequently used markers for PC but they do not allow for reliable differentiation from SCC. Nuclear protein of testis (NUT) activation and YAP1 fusion products seem to be a specific finding for PC [3]. Dermoscopic features of PC are polymorphic or atypical vessels, white globular structures and milky-red globules [3,4].

Wide local excision (WLE) and Mohs micrographic surgery (MMS) are commonly used surgical techniques for treating PC, but a consensus on optimal surgical margins remains elusive [1]. Local recurrence is a concern after WLE, with rates described as high as 20%. Local recurrence is less frequent after MMS [3]. Regarding the risk of metastasis, most articles refer to a 2017 meta-analysis of 453 patients [5]. This study reports a metastasis rate of 31%, in which metastasis to the lymph nodes and lungs was the most frequent [1,5]. It is often stated in the literature that the mortality rate after lymph node metastasis is 67%. However, many studies refer to a study which dates from 1992 [6]. To date, there are no guidelines regarding adjuvant radiotherapy or chemotherapy in metastatic disease [3]. The aim of this narrative review is to summarize the available evidence regarding epidemiology, clinical, histological and immunohistochemical diagnosis, surgical and systemic treatments, and survival rates.

## 2. Methods

**Figure 1:**
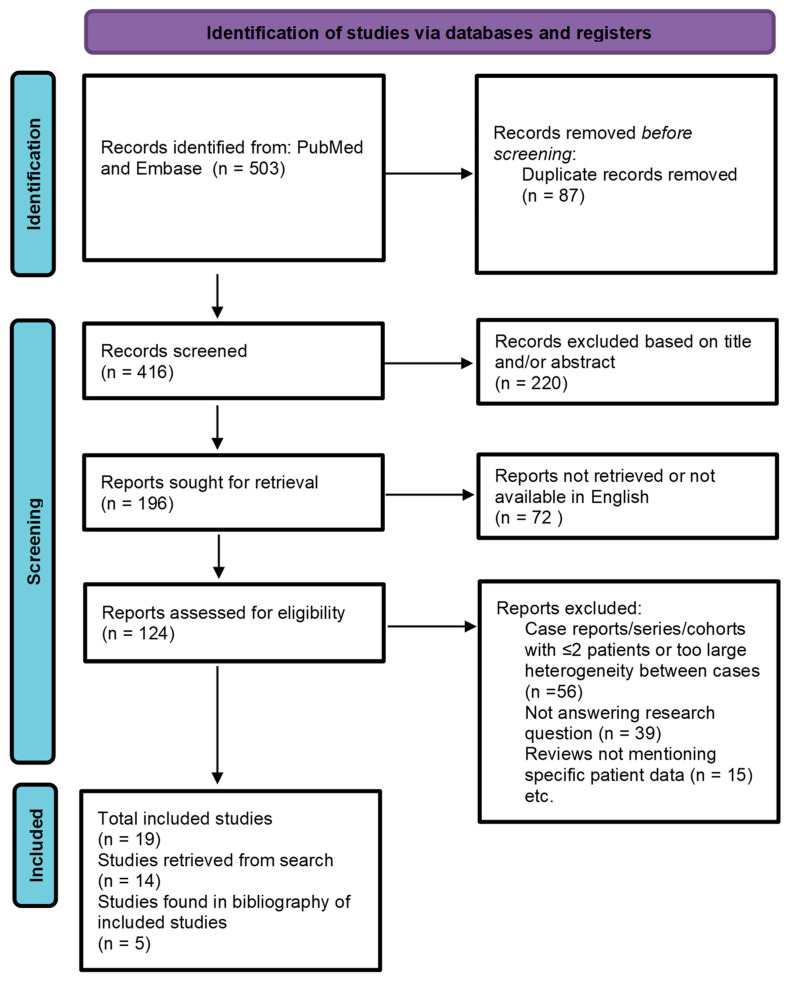
PRISMA flow diagram for performed search.

For this narrative review, a search string was used in PubMed and Embase (Figure 1). The time frame of the search was from 1 April 2012, until 26 February 2023. Search and selection of the articles were performed by one reviewer (TB). The search yielded 503 results. The language of the articles was limited to English. Outcome measures were epidemiology, clinical presentation, histopathology, immunohistochemistry, treatment, staging, survival and recurrence rates. Articles not including patient data on the above outcomes were excluded. Case reports, case series and cohorts including only 2 patients, and articles not answering our research question or not available in English were excluded. Five articles were found in the bibliography of read studies. Finally, 19 articles were included for analysis in this review. In total, the results of 2089 patients where available. De Iuliis et al. report chemotherapy in 28 patients in which other adnexal tumors are included [7]. Thus, the total number patients with confirmed PC is 2061. The screening process is shown in Figure 1. In order to create a clear, structured review, we decided to divide our outcomes into sections.

Search string:
Pubmed:

1. (“cutaneous*”[tiab] OR “cutanous*”[tiab] OR dermis[tiab] OR epiderm*[tiab]) AND (“Neoplasms, Adnexal and Skin Appendage”[Mesh] OR “porocarcinoma”[tiab])
2. Interval: 1 April 2012 until 26 February 2023

Embase:

1. (cutaneous*:ab,ti OR cutanous*:ab,ti OR dermis:ab,ti OR epiderm*:ab,ti) AND (‘neoplasms, adnexal and skin appendage’:ab,ti OR porocarcinoma:ab,ti) AND [2012–2023]/py
2. Interval 2012–2023

## 3. Results

### 3.1. Epidemiology

Porocarcinoma was mostly seen in the sixth to eighth decades, with mean age ranging between 62.1 and 82 years old (Table 1) [8,9,10,11,12,13,14,15,16,17,18,19]. The study by Salih et al., which is the largest meta-analysis on PC, involving 453 patients, reported a mean age of 67.57 years old [5]. Although more rarely, patients in the age group of 20 to 50 years old were also reported [10].

Most studies report PC to be slightly more frequent in males [8,9,10,12,13,14,16,17]. Nazemi et al. reported 52.9% males and 47.1% females [11]. However, Salih et al. (M = 49%, F = 51%), Lancelotta et al. (M = 43%, F = 57%) and Song et al. (M = 33%, F = 67%) reported a female predominance [5,18,19].

Racial distribution was reported by Nazemi et al. and Prieto-Granada et al. They showed that White patients were predominantly affected (62.5–89%). Other reported ethnicities were African American (7–10%), Japanese (16.3%), Hispanic (3%) and Asian (1%) [11,16].

### 3.2. Clinical Features

In general, PC is most frequent in the head and neck region, followed by the lower limbs (Table 1) [5,8,9,10,13,14,16,18,19]. Salih et al. reported distribution in lowering frequency on the head and neck (39.9%), lower extremity (33.9%), upper extremity (8.8%), back (5.1%), chest wall (4.6%), genitalia (4%), abdomen (2.6%) and perianal area 0.6%[5]. Nazemi et al. reported similar frequencies of tumors on the lower limb and head and neck areas, 33 and 32%, respectively [11]. Rao et al. reported a small cohort (*n* = 8) on periungual PC. They showed the hallux to be the most frequently affected digit [17]. Lancelotta et al. reported a genital distribution in 29%, which is higher than that in other studies included [19].

Morphologically, PC presents as an erythematous nodule in most cases [5,9,15,17]. Important additional features include ulceration, bleeding and plaque formation. More rarely, a verrucous, papular or nevoid form were reported [5,9]. Poroma, SCC, other CAMs, (amelanotic) melanoma and basal cell carcinoma (BCC) are the most important tumors in the differential diagnosis [3,15].

Lesions can reach substantial dimensions. Avraham et al. described tumor sizes of <2 cm in 23.6%, 2–5 cm in 10.3% and >5 cm in 31% [10]. Prieto-Granada et al. showed lesions ≥2 cm in 30% and <2 cm in 70% [16]. Yazar described a mean tumor size of 2.53 cm [8].

There is often an important diagnostic delay, with more than half of patients reporting a duration of >1 year, and a mean duration to presentation of 5.57 years has also been reported [5,15].

Dermoscopic features were extensively reported by Di Georgi et al. Three patterns were described. The first pattern (poroma like pattern) is found in 20% of lesions and consists of round-to-oval pink-red structureless areas surrounded by white-pink halos and an orange-to-yellow background. Polymorphic vessels are present and include branched vessels, dotted and linear irregular vessels. The second pattern (SCC-like pattern) is present in 40% of cases, in which a homogenic pink structureless area with polymorphic vessels including linear irregular, branched, serpentine, glomerular or hairpin vessels are seen. The lesion is also surrounded by a white halo. Overlap of patterns 1 and 2 is possible. A third pattern is present in 20% (basal cell carcinoma like pattern) and includes blue-grey globules surrounded by white areas and partially pigmented areas. Prominent arborizing vessels are present [9].

### 3.3. Diagnosis

#### 3.3.1. Histology

Only a few studies provide a detailed report of histological features (Table 1) [8,14,15]. The main features were the proliferation of median-sized cells with nuclear atypia, increased mitotic rate and apoptotic rate, mature duct formation, necrosis, ulceration, intra-cytoplasmatic lumina, and squamous differentiation. Interestingly, associated benign lesions were reported in 43.2%, of which poroma was most frequent (27%) [14]. Lymphovascular and perineural invasion are present in a minority of the cases (16% and 6.9%). Clear cells and spindle cells can also be seen. The growth patterns described are infiltrative, pushing, mixed and Bowenoid [8,14,15].

#### 3.3.2. Immunohistochemistry and Fusion Products

Rao et al. performed a systematic review on periungual neoplasms and reported eight PCs. They showed IHC positivity for PAS (13%), EMA (25%), CEA (50%) and S100 (25%) [17].

The roles of YAP1, NUT, MAML2 and WWTR1 fusion products were assessed in three studies (Table 2) [20,21,22]. Macagno et al. assessed the NUT expression in 835 cutaneous tumors and 15 specimens of normal skin. A total of 78 poroid lesions (poroma, poroid hidradenoma, dermal duct tumor, PC, malignant poroid hidradenoma) were included. NUT expression was present in 44% of benign poroid lesions and 20% of poroid neoplasms. NUT expression was present in 11% of PC and 80% in malignant poroid hidradenoma. There was no NUT expression in normal skin or other cutaneous tumors. YAP1-NUT fusion was present in all NUT-positive neoplasms (*n* = 15). Specificity was 100% for benign and malignant poroid tumors but sensitivity was only 20% for malignant poroid tumors (PC and malignant poroid hidradenoma) [21].

Kervarrec et al. examined the role of YAP1 expression, YAP1-MAML2 fusion, YAP1-NUTM1 fusion and retinoblastoma protein (RB1) expression on the diagnosis and differentiation of PC from other skin tumors like SCC and MCC (among others). Fourteen PCs were examined. In 72% of tumors, a complete loss of YAP1 expression was observed. In all YAP1-deficient tumors, a YAP1 fusion with either MAML2 (43%) or NUTM1 (29%) was present. In SCC (both in situ and invasive) and MCC, a complete loss of YAP1 expression was present in 15% and 98%, respectively. However, YAP1 fusion products could not be found in any of these tumors. The authors propose that the loss of YAP1 expression could be caused by the complete loss of RB1 expression in SCC and MCC, which is only rarely seen in PC. One sebaceoma showed a complete loss of YAP1 expression and exhibited a YAP1-MAML2 fusion [22].

Sekine et al. also confirmed the specificity of YAP1-NUTM1 and YAP1-MAML2 (or reciprocal) fusion products for poroma and PC, since these were not found in any other cutaneous tumor (including SCC, BCC, MCC, cutaneous adenocarcinoma and seborrheic keratosis), except for poroid hidradenoma. A WWTR1-NUTM1 fusion product was only found in one poroma, but not in PC. In this study, YAP1 fusions were present in 88.5% of poromas and 63.6% of PC [20].

#### 3.3.3. Stage at Diagnosis

Lymph node metastasis varied between studies from 3.7% to 21.4% [5,9,11,13,15,16]. Salih et al. reported 453 PC and reported a metastasis rate of 19.8% and 14.5% for lymph node and distant metastasis, respectively. The most frequent areas for distant metastasis were lung (12.8%), liver (9%), brain (9%) and skin (5.8%) [5]. Distant metastasis rates were lower in other studies, ranging from 1% to 16.2% [11,12,13,14,15]. AJCC stage is only seldom reported. Avraham et al. reported a cohort of 203 PC, where nearly 70% had no AJCC stage. Of the patients with known AJCC stage, 56% was stage I, 21% was stage II, 19% was stage III, and 3% was stage IV [10]. Goyal reported similar results, with 61%, 28%, 9% and 2% for stages I–IV, respectively [12]. Both authors used the AJCC 6th edition.

### 3.4. Management

#### 3.4.1. Surgical Management

Multiple studies discuss the different surgical modalities in the treatment of PC (Table 3). In general, WLE is most frequently used for primary PC. Margins are not reported by some studies [13,14,15,17]. Goyal et al. reported the largest cohort of 644 patients and performed WLE with a margin <1 cm in 76.6% of patients and a margin of >1 cm in 2.7% of patients. Other reported treatments in this cohort were punch-, shave- or incisional biopsy (19.8%), excisional biopsy (0.5%) and biopsy followed by either electrodessication, cryotherapy or laser therapy [12]. Excision margins of up to 2 cm have also been reported [16]. Mohs micrographic surgery is also frequently performed. Tolkachjov et al. reported 47 patients treated with MMS [23]. Other reports of MMS varied from 1.8% to 15.8% [9,11,14,15]. Le et al. reported the need for two stages of MMS in 63.2% [15].

In case of periungual PC, amputation was most frequently performed (62.5%) [17]. 

#### 3.4.2. Lymph Node Biopsy and Lymph Node Dissection

Sentinel lymph node biopsy (SLNB) was reported by only two studies (Table 3). Yazar et al. reported the use of SLNB in three patients when tumor depth was ≥3 mm [8]. Prieto-Granada et al. reported the use of SLNB in five patients with PC (19%) [16]. Goyal et al. reported lymph node biopsy in 50 patients, of which 18 were positive. It is, however, not specified whether these were SLNB or fine-needle aspirations in the case of clinically overt nodes [12].

In the studies included, lymph node dissection (LND) was performed in clinically overt nodes [5,11,14,15,16]. Yazar et al. also performed LND in patients with a tumor diameter >4.5 cm [8]. Nazemi et al. reported 35 patients with nodal metastasis at diagnosis. The most frequently used treatments were WLE with LND (22.8%), amputation with LND (14.3%), WLE with adjuvant radiotherapy (ART) and LND (11.4%), WLE and chemotherapy (CHT) (11.4%) and WLE and ART without LND (8.6%) [11].

#### 3.4.3. Radiotherapy and Systemic Therapies

Fionda et al. conducted a systematic review based on case reports examining the role of postoperative radiotherapy. Indications for radiotherapy were tumor subtype, tumor diameter, tumor-free margin status, invasion depth and mitotic rate. Tumors on the scalp were most frequently treated with adjuvant radiotherapy [19]. In other reports included, radiotherapy was used in a minority of the patients. Kim et al. reported adjuvant radiotherapy in 5.4% of patients [14]. Nazemi et al. only used adjuvant radiotherapy (ART) in 25.7% of patients with lymph node metastasis (*n* = 35) [11]. Le NS performed radiotherapy in 16.7% of patients, of which 5% received primary radiotherapy, 55% ART, 15% had recurrent disease and 20% had metastatic disease [15].

Only a few studies reported the use and regimens of chemotherapy. The main indications for CHT are nodal metastasis, recurrent disease and distant metastasis [7,11,15]. Chemotherapy for primary PC is only rarely used (0.6–1.8%) [11,15]. Nazemi et al. reported the use of electrochemotherapy (ECT) in 0.6% of patients with primary PC [11]. Adjuvant CHT is reported in 20% of patients with LN metastasis [11]. Le NS et al. reported adjuvant CHT in 3.6% of all patients [15]. Multiple treatments are reported for nodal disease and consist of combinations of WLE, adjuvant radiotherapy and/or CHT, WLE alone and EC with LND [11].

Miyamoto et al. reviewed 28 cases in which systemic therapy was used. Carboplatin and cisplatin were used in 60% of patients. Cisplatin was often used alongside 5-FU [3]. The use of cisplatin, carboplatin and 5-FU was confirmed by Nazemi and De Iuliis (which pooled PC with other CAMs) [7,11]. Other frequently used agents include doxorubicin, vincristine, mitomycin, bleomycin and cyclophosphamide. Monotherapy and combination therapies have been reported [7,11]. Miyamoto also reported a case in which cetuximab and paclitaxel were used in combination with radiotherapy. Two cases where pembrolizumab was used were also reported [3].

### 3.5. Outcomes

#### 3.5.1. Stage

The study of Goyal et al. stands out as the sole study providing survival rates categorized by AJCC (6th edition) stages. Their findings reveal notable distinctions in five-year disease-specific survival rates: 97.4% for stage I, 95.2% for stage II, and a substantial drop to 66.1% for stage III. Unfortunately, details on the survival rate for stage IV are either absent from their report or indicated as 0% [12].

#### 3.5.2. Surgical Treatment

Le Nguyen-Son et al. showed rates of local recurrence (LR), regional recurrence (RR) and DM after WLE and MMS in 14.7% vs. 0% (*p* = 0.084), 25.3% vs. 0% (*p* = 0.017), and 8.8% vs. 0% (*p* = 0.171), respectively, after a mean follow-up of 20 months (Table 3) [15]. Other studies confirmed that RR was higher after WLE (8–10.7%) compared to MMS (2.4%) [11,16]. Tolkachjov et al. carried out a literature study and found 47 patients treated with MMS. Only two patients developed LN metastasis, but no DM or disease-specific mortality was seen, although follow-up times were not mentioned [23].

Prieto-Granada reported local and regional recurrences, of 11% and 14%, respectively, after WLE [16]. Yazar saw no recurrences nor metastasis in their cohort (*n* = 7), of which 42% had a narrow excision (1.28 mm mean) [8].

Five-year disease specific survival (DSS) was assessed by Goyal et al. according to surgical technique. They found 5-year DSS rates of 96.3% after shave/punch/incisional biopsy with observation, 98.8% after narrow excision, 99.3% after excision with margin < 1 cm and 99.1% after excision with margin >1 cm [12]. In this study, MMS was included in the pool of patients with narrow excision and thus a direct comparison of MMS to WLE could not be made. It is to note, that 5-year overall survival was 64.5% after shave/punch/incisional biopsy, 65.2% after excisional biopsy, 69.4% after narrow biopsy and 77.7% after WLE > 1 cm margin. However, patient demographics and stage for each treatment group is not available [12].

The occurrence of LR, RR and DM had a significant impact on mortality but only the occurrence of DM had a significant impact on 3-year OS [15].

#### 3.5.3. Lymph Node Biopsy

Goyal et al. examined the role of lymph node biopsy in multiple cutaneous adnexal malignancies (CAMs). Five-year survival of patients with PC with positive nodes was 50.4%, and with negative nodes 93.8%, although the authors state no significant difference in overall survival was detected in patients with positive or negative lymph nodes for all the examined CAMs. The authors computed the positive predictive value (PPV) and negative predictive value (NPV) by consolidating the results of lymph node biopsy across all CAMs. Their analysis revealed a PPV of 0.14 and an impressive NPV of 0.98 for predicting outcomes (alive vs. deceased) specifically related to non-melanoma skin cancer [12]. As previously stated, Goyal only report lymph node biopsy and does not specify whether biopsies were SLNB or fine needle aspiration. Yazar and Prieto-Granada had a combined 7 patients were SLNB was performed, all examined nodes were negative [8,16].

#### 3.5.4. Lymph Node Dissection

The benefits of LND are unclear (Table 3). In patients with nodal disease Nazemi reported a risk of 25% for additional LN metastasis after both WLE monotherapy and WLE with LND, after 4 and 9.8 months, respectively [11]. Furthermore, they reported rates of DM in patients with nodal disease after WLE + LND, amputation + LND, WLE + LND + RT and radiotherapy alone in 50%, 20%, 50% and 50%, respectively (after 1~6.5 months) [11].

#### 3.5.5. Radiotherapy and Systemic Therapies

Fionda et al. conducted a systematic review combining 14 case reports where ART was performed. The authors reported no local or regional recurrence after radiotherapy, although DM occurred in 42.8% of patients [19]. In case of nodal disease, patients receiving WLE with LND and adjuvant radiotherapy were alive without disease in 25% after 12 months and had DM in 50% after 4.5 months. When WLE with ART, WLE with CHT or ECT with LND was performed, all patients were alive after 5–48 months [11].

Le NS reported significantly worse 1- and 3-year overall survival when CHT was used. The authors state this is possibly caused by more extensive disease in these patients [15]. Miyamoto reported either a complete or partial response after the use of cisplatin or carboplatin in 31.3%. The authors also reported a case in which cetuximab and paclitaxel in combination with radiotherapy were used, showing a complete response for 6 months [3,24]. Pembrolizumab had a complete or partial response in two cases after 16 and 18 months, respectively [3,25,26].

#### 3.5.6. Risk Factors for Adverse Outcomes

Risk of nodal or distant metastasis appears to be correlated with anatomical distribution. The risk of lymph node metastasis was highest in the genital/gluteal area, followed by the trunk, lower limb, upper limb and head and neck region. The risk of DM was also highest in the genital/gluteal area followed by upper limb, head and neck region and lower limb [11].

Le NS reported bleeding lesion, high mitotic activity, lymphovascular invasion (LVI) and the absence of surgical intervention as risk factors for DM. They also report clinical and histological ulceration and LVI as negative predictors for 3-year OS. High mitotic activity, the occurrence of DM, absence of surgical treatment and CHT were negative predictors for 1- and 3-year OS [15]. Interestingly, Di Georgi et al. found clinical ulceration to be a positive predictor for overall survival [9].

## 4. Discussion

In general, PC is a tumor of the elderly population, and most studies agree that it is most prevalent in the sixth to eighth decades, although age groups from 20 to 50 years old were also reported [10]. As previously reported, PC can arise from pre-existing poroma after a period of multiple years [1,2]. Given that the reported ages are the ages at diagnosis, it is conceivable that early onset PC can occur in younger patients as well. Regarding sex distribution, conflicting findings exist. Some studies indicate a male predominance, while others report a female predominance. With the exception of Song et al. and Rao et al., most studies only report slight differences in sex distribution [17,18]. Both studies had a limited number of patients, raising the possibility of skewed results. White patients might be at higher risk for developing PC, although this is reported in only a few studies [11,16]. It is worth noting that the study by Prieto-Granada et al. pooled demographic results with other CAMs, which could potentially influence the racial distribution [16].

Most studies agree PC presents mostly in the head and neck region and lower extremities [5,9,11,14,18]. However, our findings indicate a higher frequency in the head and neck region compared to the lower limbs, which contrasts with the existing literature [3]. UV exposure seems to be a risk factor for PC. This could be supported by the work of Harms et al. The authors reported a high mutational burden in tumors in the head and neck region with an important UV signature on a background of solar elastosis [27]. When looking at periungual PC, the hallux seems the be the most important location, although the sample size of this study was small [17].

The studies included in this analysis collectively identified nodule formation, bleeding and ulceration as typical clinical features of PC [5,9,15]. This aligns with the existing literature, although this finding is not specific.

Interestingly, there seems to be a long delay between the onset of symptoms and presentation, though this was only reported by two studies [5,15].

In our review, only one study reported dermoscopic findings found in PC. The authors outlined three distinct dermoscopic patterns, underscoring the importance of histological confirmation and cautioning against clinical diagnosis [9].

Histological details are often not extensively covered in most studies. However, in the studies incorporated into our review, the primary histological findings included mature duct formation, cytological atypia, and, in certain instances, elevated mitotic rates [8,14,15].

Intriguingly, one study noted a benign component in almost 43.2% of PC cases, with poroma being the most frequently identified. This could support the hypothesis that some PCs may develop from pre-existing poroma.

Since the differential diagnosis with SCC can be difficult, we also examined the role of immunohistochemistry [17,20,21,22]. YAP1, NUTM1 and MAML2 fusion products were shown to be highly specific for poroid neoplasms, making it a very well-performing tool to differentiate PC from SCC or MCC. Sekine et al. postulate that since YAP1-MAML2, YAP1-NUTM1 and WWTR1-NUTM1 fusion products retain their TEAD-binding domains, these fusion products may serve as a transcriptional activator of TEAD [20]. The activation of TEAD can lead to cell proliferation and growth [28,29,30]. Since these fusion products are found in poroma and PC, the role of poroma as a precursor lesion is reinforced. Thus, these fusion products could possibly also play a role in pathogenesis. A loss of YAP1 expression, without a fusion product, is not specific for poroma or PC as it could be detected in nearly all MCCs and in a minority of SCCs. PC can then be differentiated from SCC and MCC by its preserved RB1 expression [22]. Detailed information about EMA, CEA, PAS and S100 positivity was only reported by one study and it was not compared to other skin tumors. The use of these markers for PC diagnosis thus needs further examination [17].

The majority of studies reported the use of WLE for the treatment of primary PC. In one report, MMS had a clear benefit over WLE regarding LR, RR and DM [15]. Interestingly, Goyal reported excellent 5-year DSS after punch/shave/incisional biopsy only [12]. This is paradoxical since Le NS et al. found the lack of surgery to be a negative predictor for 1- and 3-year overall survival [15]. It is important to note that these survival rates were reported exclusively looking at surgical technique. Other patient demographics or disease extent were not incorporated into the survival analysis [12]. Additionally, Yazar reported no recurrence or metastasis in three patients with only narrow excision. Given the limited cohort size, conclusions cannot be drawn. In our opinion, it is advisable to obtain adequate surgical margins of at least 1 cm or use MMS for the treatment of PC.

The roles of SLNB and LND remain unclear. Goyal reported 5-year OS of 50.4% when lymph node biopsy was positive, compared to nearly 94% when nodes were negative. The authors state, however, that a difference in overall survival could not be shown. As previously stated, the authors did not specify whether this was SLNB or a fine-needle aspiration in the case of positive nodes. They did, however, report an NPV of 0.98 for predicting alive vs. dead for non-melanoma skin cancer in the case of negative lymph nodes. Importantly, this was a pooled analysis for other adnexal neoplasms [12]. To date, there is no clear consensus for indications for SLNB. We found very little data on the benefits and indications of LND. Only one study reported data of patients with various treatments for nodal disease. Overall, the follow-up times and reported outcomes varied greatly between treatments and the cohort was small. Interestingly, WLE compared to WLE with LND had a risk for additional nodal metastasis of 25% in both cases, although metastasis occurred after 4 months after WLE alone and 9.8 months after WLE combined with LND [11]. More research is needed to determine whether LND provides survival benefits.

As previously stated, many studies refer to a mortality risk of 67% in the case of nodal disease [6]. Goyal et al. found a 5-year disease-specific survival of 66.1% in the case of deep dermal invasion or nodal metastasis (AJCC 6 stage III); thus, mortality risk seems to be lower than previously reported [12].

There is very little evidence regarding the use of CHT and radiotherapy. Although no consensus exists, the choice for the use of radiotherapy is based on clinical and pathological features and positive margins. Only one systematic review is included in our report, which only reports 14 patients. The authors suggest that all patients need to be discussed on a multidisciplinary board [19].

The administration of chemotherapy exhibited significant heterogeneity both between and within the studies that were reported. Most studies do not report that chemotherapy regimens were used, and, within studies, the use of CHT varies greatly between the reported patients. Overall, the main indications seem to be nodal disease and DM. Prospective studies are needed to determine the role and regimens of CHT for PC treatment. Miyamoto et al. reported promising results from case reports in which cetuximab and pembrolizumab were used [3,24,25,26]. This is confirmed by Westphal et al., who used a cell line of metastatic PC to investigate systemic treatments. They found high EGFR and PDL1 expression in tumoral tissue. Therapy with cetuximab followed by nivolumab was started, after which disease progression occurred after 7 and 5 months, respectively. MEK inhibitors also inhibited growth and induced apoptosis in the examined PC cell line, making it a potential therapeutic target [31]. In the last decade, the treatment of melanoma and non-melanoma skin cancer has changed drastically with the implementation of immunotherapies. Since skin tumors have a high mutational burden, they are sensitive to immune checkpoint inhibition [32]. We suspect that the use of these therapies in advanced PC could benefit patients with advanced disease. Overall, prospective studies are needed to determine the systemic treatment for PC.

## 5. Conclusions

We conclude that PC is a very rare cutaneous tumor mainly occurring in the elderly in the head and neck region and in the lower limbs. Diagnostic delay remains an important problem. The risk for both nodal and distant metastasis is high. Mohs micrographic surgery seems promising in surgical treatment. Further studies are needed regarding benefits and indications of SLNB, LND, radiotherapy and systemic therapies. We suggest all cases of PC to be referred to tertiary care hospitals so prospective studies can be conducted.

## Figures and Tables

**Table 1:** Demographic, clinical and pathological features.

Author	*n*	Age (Years) and Sex	Clinical Features	Pathology
De Giorgi [9]	52	Mean age 82 (49–96)Male 59.6% Female 40.4%	Head and neck region (47%), lower limb (25%), trunk (15%) Erythematous nodule (64%), ulcerated (50%) Dermoscopic patterns: poroma-like (20%), SCC-like (40%), BCC-like (20%)	Ulceration present in 58%
Goyal [12]	644	Median age 72 (20–103) Male 56.5% Female 43.5% White 82.8%	NS	NS
Kim H [14]	37	Mean age 65.6 ± 16.4 Male 51% Female 49%	Head and neck (29.7%), trunk (27%), lower limb (24.3%), pelvis (8.1%)	Nuclear atypia Increased mitosis ± apoptosis Medium sized cells Mature duct formation (66.7%) Necrosis (66.7%) Benign lesion associated in 43.2% of which poroma is 27% Squamous differentiation (55.6%)
Avraham [10]	203	Age: 20–50 (13.8%) ≥70 (59.1%) Male 56.7% Female 43.4%	Head and neck (27.6%), trunk (18.7%), limbs (15.8%), NS (37.9%) Size: ≤2 cm (23.6%), 2–5 cm (10.3%), >5 cm (31%), NS (35%)	NS
Salih [5]	453	Mean age 67.6 Male 49% Female 51%	Head and neck (39.9%), lower limb (33.9%), upper extremity (8.8%), other (16.9%) Mass/nodule (71%), ulceration (18%), plaque (9.8%) Mean duration to presentation 5.57 years	NS
Le NS [15]	120	Age: <51% (17,5%), ≥51 (82.5%)	Only head and neck PC included, scalp most frequent (35.8%). Nodule (33.3%), ulceration ± bleeding (31.7%), erythematous (15.8%) Duration to presentation >1 year in 55.6%	Nuclear pleomorphism (59.4%) Increased mitotic rate (68.9%) Duct formation (81%) Ulceration (29.7%) Necrosis (35.4%) Perineural invasion (6.9%) Lymphovascular invasion (16%) Growth patterns: Infiltrative: 52.2% Pushing 34.8% Mixed 13.0%
Rao [17]	8	Mean age 72.9 Male 88% Female 12%	Periungual PC: - hallux in 50% - finger IV in 25% Erythematous tumor, telangiectasia, plaque formation, nail abnormalities	/
Prieto-Granada [16]	28	Mean age 61 Male 61% Female 39% White 89% African American 7% Hispanic 3% Asian 1%	Pooled with other CAMs: Head and neck (55%), limbs (29%), trunk (16%) ≥2 cm in 30%	NS
Nazemi [11]	206	Mean age 63.6 Male 52.9% Female 47.1% White 62.5% Japanese 16.3% African American 10%	Lower limb (33%), head and neck (32%), trunk (14.7%), upper limb (7.4%), perigenital (11.2%)	NS
De Georgi [13]	133	Mean age: ≤69: 24% ≥70: 76% Male 50.4% Female 49.6%	Face (33%), scalp (20%), lower limbs (17%), upper limbs (11%)	NS
Yazar [8]	7	Mean age 62.1 Male/female ratio: 1:0.4	Face (42%), back (28%), inguinal (14%), scalp (14%) Mean tumor size 2.53 cm	Atypical cells 85% Increased mitotic rate 85% Necrosis 57% Mean thickness 3.06 cm
Fionda [19]	14	Mean age 63 Male 43% Female 57%	Head and neck (43%), genitalia (29%), lower limb (21%), upper limb (7%)	NS
Song [18]	21	Mean age 66 Male 33% Female 67%	Head and neck (38%), lower limb (33%), trunk (14%), upper limb (4.8%)	NS

CAM: cutaneous adnexal malignancy, SCC: squamous cell carcinoma, BCC: basal cell carcinoma, NS: not specified.

**Table 2:** Immunohistochemistry.

Author	*n*	Immunohistochemical Findings
Macagno [21]	78 poroid lesions 835 cutaneous tumors	NUT expression: benign poroid tumors (44%), PC (11%), MPH (80%), other cutaneous tumors (0%) Sensitivity: 20% for malignant poroid lesions (PC and MPH) Specificity: 100% for benign and malignant poroid lesions YAP1-NUTM1 fusion products present in 100% of tested (*n* = 12) NUT positive neoplasms
Kervarrec [22]	14	YAP-NUTM1 fusions 29% YAP-MAML2 fusions 43% Loss of YAP1 expression possible in SCC and MCC, fusion products are not present Complete loss of Rb expression in YAP1 deficient tumors: frequent in SCC and MCC, rare in PC
Sekine [20]	11	PC: (*n* = 11) YAP1-MAML2: 9% YAP1-NUTM1: 55% Poroma: (*n* = 104) YAP1-MAML2: 68% MAML2-YAP1: 46% YAP1-NUTM1: 20% WWTR1-NUTM1: 1%

SCC: squamous cell carcinoma, MCC: Merkel cell carcinoma, PC: porocarcinoma, MPH: malignant poroid hidradenoma.

**Table 3:** Surgical treatment and outcomes.

Author	*n*	Surgical Management	Outcomes
Goyal [12]	644	Different interventions: Punch/shave/incisional: 19.8% Excisional biopsy: 0.5% Biopsy followed by electrodessication/cryosurgery/laser: 0.5% Excision < 1 cm margin: 76.6% Excision > 1 cm margin: 2.7%	5-year disease specific survival: 1. 96.3% 2. 98.8% 4. 99.3% 5. 99.1%
Rao [17]	8	Periungual PC - Amputation: 62.5% - Excision 37.5%	Recurrence: 25% (not further specified)
Prieto-Granada [16]	28	WLE (1–2 cm margin) in all patients SLNB: 19% (0% positive) LND: 21% (in case of positive nodes)	Local recurrence: 11% Regional recurrence: 14%
Nazemi [11]	206	Primary PC: (*n* = 160) - WLE: 55% (1 cm margin) - MMS 26.2% - Amputation 2.5% LN disease: (*n* = 35) - WLE: 11.4% - WLE + LND: 22.8% - Amputation + LND 14.3% - WLE + LND + ART: 11.4% - WLE + ART: 8.6% - WLE + ACT: 11.4% - WLE + ACT + ART: 5.7% - Amputation 2.9% - ECT + LND 2.9%	Primary tumors: - WLE compared to MMS: ○ AWOD: 56.8% vs. 83% ○ LN metastasis: 11.4% vs. 2.4% Nodal metastasis: - WLE: ○ AWOD (180 months) in 25%, additional LN metastasis in 25% (4 months) - WLE + LND ○ DM in 50% (6.5 months), additional LN metastasis in 25% (9.8 months) - Amputation + LND: ○ AWOD in 40%, DM in 20% (7 months both) - WLE + LND + ART ○ AWOD (12 months) in 25%, DM in 50% (4.5 months) - WLE + ART/WLE + CHT/ECT + LND - : AWOD 100% (5–48 months)
Yazar [8]	7	Excision: - Single stage: 42% (mean margin of 1.28 mm) - Double stage: 57% (mean margin 8.83 mm) SLNB: 28% of patients (when tumor dept ≥3 mm) LND: 14% (tumor diameter >4.5 cm)	No positive nodes at diagnosis Follow up (mean 36 months) - No recurrences - No metastasis
Kim [14]	37	WLE: 64.9% WLE + ART: 5.4% WLE + LND + ACT: 2.7% MMS: 5.4%	No outcomes after surgery reported
Le NS [15]	116	WLE: 76.7% MMS: 15.8% (2 stages in 63.2%) LND: 15% (of which 72.2% at diagnosis and 27.8% at recurrence) Radiotherapy: 16.7% - Primary 5% - Adjuvant: 55% - Recurrence: 15% - Metastasis): 20% CHT: 10.8% - Primary: 15.2% - Adjuvant: 30.8% - Recurrence: 23.1% - Metastasis: 23.1%	Local recurrence: (*p* = 0.084) - WLE: 14.7% - MMS: 0% Regional recurrence: (*p* = 0.017) - WLE: 25.3% - MMS: 0% DM (*p* = 0.181) - WLE: 8.8% - MMS: 0% Significantly higher mortality rate after local and regional recurrence and metastasis. Only significant difference in 3-year OS for DM.
Tolkachjov [23]	46	MMS	After MMS: Nodal metastasis: 4.2% Local recurrence, DM and disease specific mortality 0%
De Georgi [13]	133	Surgery: 99% (margins not specified) CHT: in selected cases (indications not specified)	Local recurrence: 2.3% after 48.7 months.

WLE: wide local excision, SLNB: sentinel lymph node biopsy, LND: lymph node dissection, MMS: Mohs micrographic surgery, ART: adjuvant radiotherapy, ACT: adjuvant chemotherapy, ECT: electrochemotherapy, CHT: chemotherapy.
